# Effects of *Trichinella spiralis* and its serine protease inhibitors on intestinal mucosal barrier function

**DOI:** 10.1186/s13567-024-01446-z

**Published:** 2025-01-11

**Authors:** Ruibiao Wang, Yuheng Zhang, Zhixin Li, Jingbo Zhen, Qiankun Li, Qi Zhang, Yuqi Yang, Xueting Liu, Yixin Lu

**Affiliations:** 1 Heilongjiang Provincial Key Laboratory of Zoonosis, College of Veterinary Medicine, Northeast Agricultural University, Harbin, China; 2 Henan Provincial Animal Pathogens and New Veterinary Drugs, College of Animal Science and Technology, Henan Institute of Science and Technology, Xinxiang, China

**Keywords:** *Trichinella spiralis*, serine protease inhibitors, intestinal barrier, inflammation

## Abstract

*Trichinella spiralis* (*T. spiralis*) is a highly pathogenic zoonotic nematode that poses significant public health risks and causes substantial economic losses. Understanding its invasion mechanisms is crucial. This study explored how the serine protease inhibitors (SPIs) secreted by *T. spiralis* affect the host’s intestinal epithelial barrier. Furthermore, the effects of *T. spiralis* infection on the jejunal barrier function in mice were investigated. The histopathological analysis indicated significant damage to the jejunum, which peaked at day 7 post-infection (dpi). The results of RT-qPCR and western blotting revealed marked reductions in tight junction proteins (ZO-1, occludin, claudin-3), mucins (MUC-1, MUC-2), and anti-inflammatory cytokines (TGF-β, IL-10) from 1 to 15 dpi. There was also increased expression of Toll-like receptors (TLR-1, TLR-2, TLR-4) and pro-inflammatory cytokines (TNF-α, IL-1β). Recombinant SPIs (rKaSPI, rAdSPI) were purified, co-cultured with intestinal epithelial cells (IPECs), and used in mouse models. The protein expression changes in IPECs and mice were consistent with those in *T. spiralis*-infected tissues. Both SPIs caused the down-regulation of ZO-1, occludin, claudin-3, MUC-1, MUC-2, TGF-β, and IL-10 while up-regulating TLR-4 and pro-inflammatory cytokines. As a result, the intestinal barrier was disrupted, and inflammation was exacerbated. Notably, rAdSPI had a more pronounced effect. In summary, *T. spiralis* infection caused significant jejunal damage and disrupted the intestinal barrier. *T. spiralis*-secreted SPIs, especially serpin-type serine protease inhibitors (AdSPI), were pivotal in facilitating invasion by compromising the host’s intestinal barrier and promoting inflammation. This study provides insights into *T. spiralis* invasion mechanisms and the potential targets for trichinellosis prevention and control.

## Introduction

*Trichinella spiralis* (*T. spiralis*) is a highly pathogenic, zoonotic parasitic nematode that is a significant foodborne pathogen capable of parasitising over 150 animal species worldwide [1]. Infection caused by *T. spiralis* severely threatens public health, significantly impacts the livestock industry, particularly swine production, and results in substantial economic losses [2, 3]. Therefore, researching the interaction mechanisms between *T. spiralis* and its host is essential.

When muscle tissues containing encapsulated *T. spiralis* muscle larvae are ingested, the released larvae penetrate the intestinal epithelial barrier and invade the intestinal epithelial cells (IECs), developing into adult worms [4, 5]. Consequently, the intestinal epithelium barrier serves as a natural defence against *T. spiralis* infection and represents the initial site of mutual interaction between *T. spiralis* and its hosts [6, 7]. This process determines whether *T. spiralis* establishes a parasitic relationship with the host. However, the underlying mechanisms of larval invasion remain poorly understood.

Research indicates that during the intestinal phase of infection, *T. spiralis* muscle larvae engage in an immunological dialogue with the host by releasing excretory-secretory products (ESPs) to ensure their survival [8]. The excretory-secretory (ES) antigens produced by *T. spiralis* are the first substances that encounter the host immune system. They interact with a range of host cells, such as IECS and immune cells [9]. ES antigens also regulate the host’s immune response or influence the gene expression of host cells, thereby creating a suitable environment for their survival [10].

ES products comprise functional proteins that play distinct roles in inducing host immune responses, including proteases, protease inhibitors, heat shock proteins, phosphatases, and endonucleases [11, 12]. In particular, serine protease inhibitors (SPIs) are significant components of ES products [13]. SPIs not only act as inhibitors of protease activity but also serve important functions in numerous biological processes, including coagulation, complement activation, and inflammation [14, 15]. The SPIs in parasites contribute to their survival by modulating the host’s immune response [16]. Previous studies have demonstrated that SPI derived from *T. spiralis* can activate endoplasmic reticulum stress, leading to cell apoptosis and the activation of the NF-κB signalling pathway to promote the occurrence and development of inflammation [17].

Based on the above considerations, the main objective of this study was to analyse whether SPIs secreted from *T. spiralis* adult worms and larvae could influence intestinal epithelial barrier function, thereby creating a favourable environment for intestinal colonisation. This research aimed to provide insights into the invasion mechanisms of *T. spiralis* and contribute to developing effective strategies for preventing and controlling trichinellosis.

## Materials and methods

### Animal, cell, and parasite

Specific pathogen-free (SPF) female BALB/c and Kunming (KM) mice, aged 6–8 weeks, were purchased from Harbin Medical University. All animal experiments and experimental procedures followed the Regulations for the Administration of Laboratory Animals in China and the animal experimental standards approved by the Animal Management Committee of Northeast Agricultural University. All mice had ad libitum access to food and water and were maintained under SPF conditions with a humidity of 70 ± 10% and a temperature of 20 ± 2 °C.

The Harbin Veterinary Research Institute donated porcine small intestinal epithelial cells (IPECs), which were cultured in 90% DMEM medium supplemented with 10% foetal bovine serum (FBS) (PAN, Germany) and 1% penicillin/streptomycin (100 U/mL).

*T. spiralis* (ISS533), originally obtained from swine in Heilongjiang Province, China, was propagated and preserved by our laboratory through serial passage in Kunming mice. Infective larvae were isolated from the muscles of infected KM mice using the standard method [18].

### Recombinant protein preparation

KaSPI, *T. spiralis* adult worm-secreted SPI, has been assigned the GenBank accession number XM_003379851. AdSPI, *T. spiralis* muscle larvae-secreted SPI, has been assigned the GenBank accession number EU263307.1.

In our preliminary laboratory work, the recombinant proteins, pET-30a-*Ts*KaSPI (rKaSPI) and pET-28a-*Ts*AdSPI (rAdSPI) were successfully constructed [19]. The proteins were expressed in *E. coli*, purified and renatured through a nickel column per the manufacturer’s protocol (Solarbio, China). The recombinant proteins were verified by 12% SDS-PAGE, followed by Coomassie blue staining. The protein concentrations were determined using a bicinchoninic acid (BCA) kit (Meilunbio, China). The contaminated endotoxin was removed by a ToxOut™ High Capacity Endotoxin Removal Kit (GenScript Bio, China).

### Animal grouping and treatment

To induce *T. spiralis* infection, each BALB/c mouse was orally infected with 300 larvae of *T. spiralis* on day 0. Following the life cycle of *T. spiralis*, six mice were randomly selected on days 1, 3, 5, and 7 post-infection for small intestinal tissues (duodenum, jejunum, and ileum) collection and preparation for further analysis. To analyse the effects of recombinant proteins on the intestinal epithelial barrier, 24 BALB/c mice were randomly divided into four groups: blank control (no treatment, to exclude the potential impact of personal operational errors), PBS, rKaSPI, and rAdSPI. Each mouse in the protein groups received an intraperitoneal injection with 50 μg of the respective protein and small intestinal tissues; serum was collected at 3 dpi. Mice in the PBS group were intraperitoneally injected with PBS as the negative control. The infection dose of *T. spiralis* larvae and the intraperitoneal injection concentration of the recombinant protein were determined using previously established methods [17].

### Haematoxylin and eosin staining and immunohistochemistry

The small intestine tissues were fixed in 4% paraformaldehyde for 24 h, dehydrated and embedded in paraffin. They were then sectioned at a thickness of 4 μm, stained with haematoxylin and eosin (H&E), dehydrated again, and mounted for microscopic observation.

For immunohistochemical (IHC) staining, 4 μm-thick duodenal tissue sections underwent antigen retrieval in 0.01 M citrate buffer (pH 6.0) at 121 °C for 5 min. Endogenous peroxidase activity was quenched with 3% hydrogen peroxide (H_2_O_2_) for 15 min. Subsequently, the samples were incubated with 1% bovine serum albumin for 20 min at room temperature to block non-specific binding. The sections were then incubated overnight at 4 °C with the primary antibody (1:100; Wanleibio, China), followed by further incubation at 37 °C for 30 min with HRP-conjugated goat anti-rabbit antibodies (1:500; ABclonal, USA). Visualisation was achieved using 3,3ʹ-diaminobenzidine (DAB; Sigma-Aldrich, USA) staining and counterstaining with Mayer’s haematoxylin solution (Sigma-Aldrich, USA).

### RT-qPCR

Quantitative real-time PCR (RT-qPCR) was performed to detect the relative expression of genes. Total RNA was isolated from jejunal tissue or IPEC cells with Trizol Reagent (Invitrogen, USA) and then reverse transcribed into first-strand cDNA using the PrimeScript 1st Strand cDNA Synthesis Kit (Takara, Japan). Sangon Biotech, China, designed and synthesised all mRNA primers (shown in Tables 1 and 2).

**Table 1:** **Primers of the detected genes in Jejunal tissue**.

Gene name	Primers	Sequence	Access number
GAPDH	Forward	5ʹ-GTGAAGGTCGGTGTGAACGGATT-3ʹ	NM_001289726.2
	Reverse	5ʹ-GGTCTCGCTCCTGGAAGATGGT-3ʹ	
IL-1β	Forward	5ʹ-AATCTCGCAGCAGCACATCAAC-3ʹ	XM_006498795.5
	Reverse	5ʹ-TGTTCATCTCGGAGCCTGTAGTG-3ʹ	
TNF-α	Forward	5ʹ-GCCACCACGCTCTTCTGTCTAC-3ʹ	NM_013693.3
	Reverse	5ʹ-GGCTACAGGCTTGTCACTCGAA-3ʹ	
TGF-β	Forward	5ʹ-CCGCAACAACGCCATCTATGAG-3ʹ	XM_036152883.1
	Reverse	5ʹ-ACCAAGGTAACGCCAGGAATTG-3ʹ	
IL-10	Forward	5ʹ-GGGTTGCCAAGCCTTATCGGAA-3ʹ	NM_010548.2
	Reverse	5ʹ-CTGCTCCACTGCCTTGCTCTTA-3ʹ	
ZO-1	Forward	5ʹ-AAGGCGGATGGTGCTACAAGTG-3ʹ	NM_009386.3
	Reverse	5ʹ-GGCTCAGAGGACCGTGTAATGG-3ʹ	
ZO-2	Forward	5ʹ-GGCGGCTGCTGTATCGGTTA-3ʹ	NM_001198985.2
	Reverse	5ʹ-GGCGTGCTTGTCCTGCTCAA-3ʹ	
claudin-3	Forward	5ʹ-CCTTCATCGGCAGCAGCATCAT-3ʹ	NM_009902.4
	Reverse	5ʹ-GCCAGCAGCGAGTCGTACATT-3ʹ	
occludin	Forward	5ʹ-TTGGCTACGGAGGTGGCTATGG-3ʹ	NM_008756.2
	Reverse	5ʹ-AGGAAGCGATGAAGCAGAAGGC-3ʹ	
TLR1	Forward	5ʹ-ACTATGCTGGTGCTGGCTGTCA-3ʹ	NM_030682.2
	Reverse	5ʹ-TGTGCCTGGTCTGTGTCCACTG-3ʹ	
TLR2	Forward	5ʹ-AGACGCTGGAGGTGTTGGATGT-3ʹ	XM_006501460.4
	Reverse	5ʹ-AAGTGGTTGTCGCCTGCTTCC-3ʹ	
TLR4	Forward	5ʹ-GCCATTGCTGCCAACATCATCC-3ʹ	XM_036163964.1
	Reverse	5ʹ-ACAATTCCACCTGCTGCCTCAG-3ʹ	
MUC-1	Forward	5ʹ-CCTGCTGGTGCTGGTCTGTATT-3ʹ	NM_013605.2
	Reverse	5ʹ-GTAGCGTCCGTGAGTGTGGTAG-3ʹ	
MUC-2	Forward	5ʹ-CGACACTCAGCACACCAACCAA-3ʹ	NM_023566.4
	Reverse	5ʹ-CAGCGGCACAATCTCCACTACG-3ʹ

**Table 2:** **Primers of the detected genes in IPEC-J2 cells**.

Gene name	Primers	Sequence	Access number
GAPDH	Forward	5ʹ-GGTGAAGGTCGGAGTGAACG-3ʹ	NM_001206359.1
	Reverse	5ʹ-CCGTGGGTGGAATCATACTGG-3ʹ	
IL-1β	Forward	5ʹ-AAAGATAACACGCCCACCC-3ʹ	NM_214055.1
	Reverse	5ʹ-GGAGTTTCCCAGGAAGACG-3ʹ	
TNF-α	Forward	5ʹ-GCTCTTCTGCCTACTGCACTTC-3ʹ	NM_214022.1
	Reverse	5ʹ-GCTGTCCCTCGGCTTTGA-3ʹ	
TGF-β	Forward	5ʹ-ATTTACTTACTGAGCATCTTGGACCTTA-3ʹ	NM_214015.2
	Reverse	5ʹ-GGGTGTTATCAGAGTCCCTTTTAGC-3ʹ	
IL-10	Forward	5ʹ-GACTCAACGAAGAAGGCACAG-3ʹ	NM_214041.1
	Reverse	5ʹ-GCAGGCTGGTTGGGAAGT-3ʹ	
ZO-1	Forward	5ʹ-AGGTGCTCCCATCGT-3ʹ	XM_021098827
	Reverse	5ʹ-TTTCGGTAATACTCTTCATC-3ʹ	
ZO-2	Forward	5ʹ-GGAGCATTGACCCGACTTAC-3ʹ	NM_001206404
	Reverse	5ʹ-AGACCATACTCTTCATTCGCTTT-3ʹ	
claudin-3	Forward	5ʹ-CAGAGCCGTTCGCAACCAGG-3ʹ	NM_001160075.1
	Reverse	5ʹ-CACCACGCAGTTCATCCACAGG-3ʹ	
occludin	Forward	5ʹ-CACCCAGCAACGACA-3ʹ	NM_001163647
	Reverse	5ʹ-ATAACGAGCATAGACAGAAT-3ʹ	
TLR1	Forward	5ʹ-CTCTGCTCAAGGACTTCCGTGTA-3ʹ	NM_001031775.1
	Reverse	5ʹ-AGAGCCAGTGCCAGCCCAGT-3ʹ	
TLR2	Forward	5ʹ-GGTCCGATGCTGGTCTTTATC-3ʹ	NM_213761.1
	Reverse	5ʹ-GCAAGTCACCCTTTATGTTATTCA-3ʹ	
TLR4	Forward	5ʹ-CCAGTGCTGCTTTGAATAGAG-3ʹ	NM_001293316.1
	Reverse	5ʹ-GAACAGAAGTGACCCGGAGA-3ʹ	
MUC-1	Forward	5ʹ-ATGAGCTGGGAGCACAGGTGG-3ʹ	XM_001926883.5
	Reverse	5ʹ-CCAGGCTCGGATGGACTTCG-3ʹ	
MUC-2	Forward	5ʹ-AGGACGACACCATCTACCTCACTCA-3ʹ	XM_013989745.1
	Reverse	5ʹ-GCAAGGCCAGCTCGGGAAT-3ʹ	

The RT-qPCR was executed according to the instructions of ChamQ Universal SYBR qPCR Master Mix (Vazyme, China) using the Roche Light Cycler 480 system. Glyceraldehyde-3-phosphate dehydrogenase (GAPDH) was selected as a reference gene, and the results were calculated using the 2^−∆∆Ct^ method [20].

### Western blotting

The total protein used for western blotting assays was prepared from jejunal tissue or IPEC cells; protein concentration was determined with a BCA assay kit. An equal amount of protein was separated by using 12% sodium dodecyl sulfate/polyacrylamide gel electrophoresis (SDS-PAGE) and electrotransferred onto a 0.45 μm polyvinylidene fluoride (PVDF) filter membrane (Millipore, USA). The PVDF membrane was blocked in 5% non-fat milk for 2 h at room temperature and incubated with the primary antibody (Wanleibio, China) overnight at 4 ℃.

Subsequently, the PVDF membrane was washed using phosphate-buffered saline solution (PBST) and incubated with the horseradish peroxidase (HRP)-conjugated secondary antibody (ABclonal, USA) for 2 h at room temperature. Lastly, the blot was developed with the ultrasensitive enhanced chemiluminescence (ECL) reagent (Meilunbio, China), and the membrane was exposed. The bands were quantified using a chemiluminescence imaging system (Syngene, USA) and analysed with Image J software.

### Immunofluorescence

After treatment, the IPEC cells were washed with PBS and fixed with 4% paraformaldehyde for 30 min. Next, the cells were permeabilised using 0.25% TritonX-100 for 10 min and blocked with 2% bovine serum albumin for 30 min at room temperature. The cells were subsequently rinsed with PBS and incubated overnight at 4 °C with primary antibodies. The wells were washed with PBS and incubated with FITC-labelled secondary antibodies (Bioss, China) for 1 h at room temperature with no light exposure. The cell nuclei were stained with 2 μg/mL DAPI for 8 min, also in the dark. Finally, the cells were examined under a fluorescence microscope (Syngene, USA).

### ELISA

Mouse or pig double-antibody sandwich enzyme-linked immunosorbent assay (ELISA) kits (Mlbio, China) were used to measure the cytokine content in mouse serum or cell culture supernatant, respectively. Briefly, the microwells were incubated with either the experimental or standard samples at 37 °C for 30 min, followed by the addition of HRP-labelled antibodies.

The microwells were then thoroughly washed and stained with the substrate 3,3′,5,5′-Tetramethylbenzidine (TMB) for 10 min at 37 °C. The stop solution was added to terminate the enzyme–substrate reaction. The absorbance, at 450 nm, was measured using a plate reader (BioTek, USA).

### Statistical analyses

All experimental data were expressed as the mean ± SD using SPSS 22.0 software. Statistical significance was assessed using one-way analysis of variance (ANOVA) and post hoc Duncan’s test. A *P-*value of < 0.05 was considered statistically significant. The quantification of the protein band intensities was analysed using ImageJ software.

## Results

### Effects of *T. spiralis* on jejunal tissue

In our previous study [17], the highest number of worms occurred in the host jejunum following *T. spiralis* infection. Consequently, the jejunum was selected as the focus of this study. In consideration of the life cycle of *T. spiralis*, jejunum samples were collected post-infection for H&E pathological section analysis on 1 dpi (larvae developing into adults), 3 dpi (adult mating), 7 dpi (numerous newborn larvae), and 15 dpi (newborn larvae migration). The scoring system, referenced and modified from Fernández-Blanco et al. [21], was employed for double-blinded pathological scoring according to the criteria presented in Table 3.

**Table 3:** **Histopathological scoring**.

Indicators	Score			
	0	1	2	3
Epithelial cell status	No change	Mild	Moderate	Severe
Villus integrity	No change	Mild	Moderate	Severe
Central lacteal expansion	No change	Mild	Moderate	Profound
Leukocyte infiltration	< 10 Leukocytes per field	11–15 Leukocytes per field	16–20 Leukocytes per field	> 20 Leukocytes per field
Submucosal oedema	No change	Mild	Moderate	Profound
Mucosal hyperaemia	None	Mild	Moderate	Severe
Number of goblet cells	< 3 Goblet cells per field	3–5 Goblet cells per field	6–10 Goblet cells per field	> 10 Goblet cells per field

Normal epithelial cells are columnar, closely arranged, monolayer intestinal epithelial cells; Normal villus has an intact morphology, no break, and no shedding.

**Figure 1:**
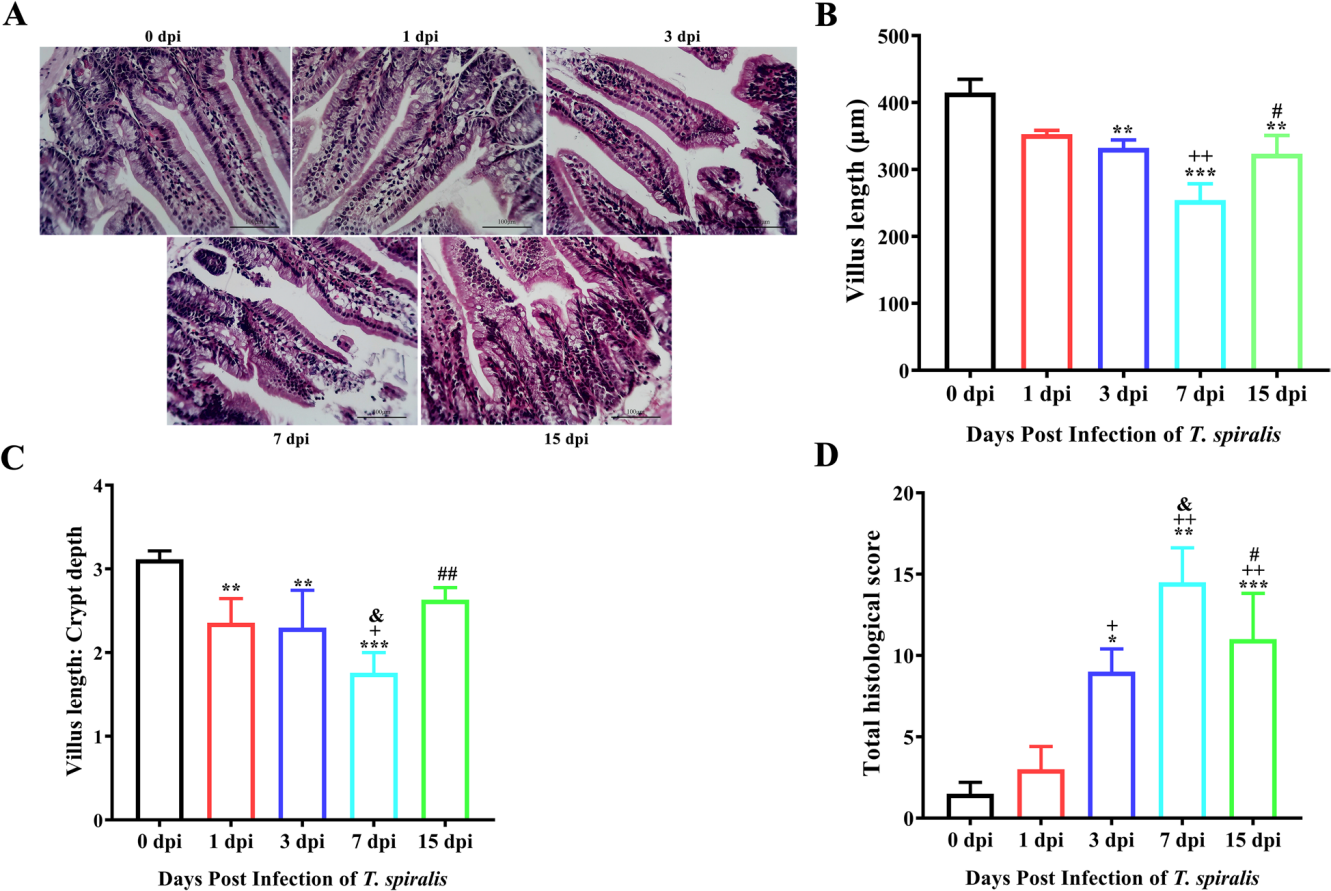
**H&E staining for evaluating pathological injury of the jejunum**. Histopathological sections of the jejunum were collected from mice on Days 1, 3, 7, and 15 post-infection. **A** H&E staining of the jejunum. Magnification, × 200. **B** Villus length of the jejunum. Villus length and crypt depth of the jejunum were measured using Image-Pro Plus software. **C** The ratio of villus length/crypt depth. **D** The intestinal pathology scores. The data were expressed as the mean ± SD. *P < 0.05, **P < 0.01, ***P < 0.001 compared with the 0 dpi group; +*P* < 0.05, ++*P* < 0.01, +++*P* < 0.001 compared with the 1 dpi group; &*P* < 0.05, &&*P* < 0.01, &&&*P* < 0.001 compared with the 3 dpi group; #*P* < 0.05, ##*P* < 0.01, ###*P* < 0.001 compared with the 7 dpi group.

The histopathological results indicated that on 3 dpi, *T. spiralis* infection caused significant jejunal damage, characterised by disorganised intestinal cell arrangement, fragmented and sloughing villi, extensive inflammatory cell infiltration in the lamina propria and submucosa, and an increased number of goblet cells. On 7 dpi, the damage was more severe; however, on 15 dpi, the pathological scores decreased compared to the scores at 7 dpi (*P* < 0.05) (Figures 1A and D). Furthermore, the assessments of jejunal villus length and the villus/crypt ratio revealed significant declines in both metrics on 3 dpi, with the most severe damage evident on 7 dpi (Figures 1B and C). H&E pathological sections established that *T. spiralis* infection induced the most pronounced jejunal tissue damage on 7 dpi.

### Effects of *T. spiralis* on host intestinal barrier function

To examine further the influence of *T. spiralis* infection on the intestinal mucosal barrier, RT-qPCR and western blotting were utilised to analyse the expression of key barrier-related factors, including tight junction (TJ) proteins, mucins, Toll-like receptors (TLRs), and inflammatory cytokines.

**Figure 2:**
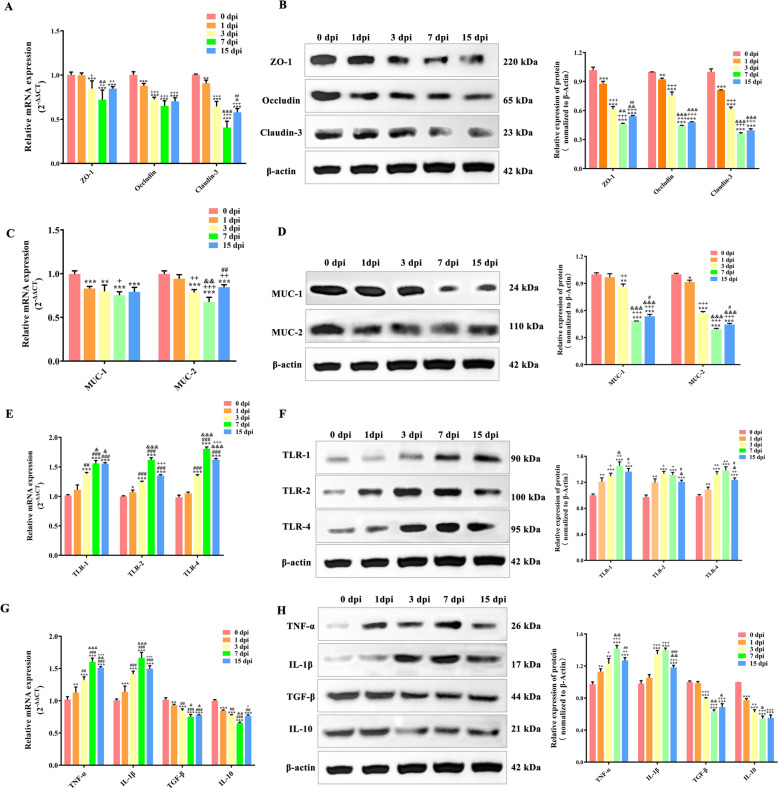
**Effects of** ***T. spiralis* on host intestinal barrier function**. Protein and mRNA acquisition in the jejunum of mice infected with *T. spiralis* on dpi 0, 1, 3, 7, 15. Verification of expression changes of TJ proteins (**A**, **B**), Mucin (**C**, **D**), TLRs (**E**, **F**), and inflammatory cytokines (**G**, **H**) by RT-qPCR and western blot, respectively. All assays were performed in triplicate, and data were presented as the mean ± SD. Significance markers were the same as those in Figure 1.

RT-qPCR analysis indicated a marked decline in the TJ protein ZO-1 within the jejunum at 1 dpi (*p* < 0.001) compared to the uninfected group (at 0 dpi). Furthermore, occludin and claudin-3 expressions underwent significant reductions on 3 dpi (*P* < 0.001), reaching their lowest levels on 7 dpi. Notably, despite a noteworthy up-regulation of ZO-1, occludin, and claudin-3 on 15 dpi in comparison to 7 dpi, these levels remained significantly inferior to those on 0 dpi (Figure 2A), highlighting the persistent effects of *T. spiralis* infection on the intestinal mucosal barrier.

The western blot analysis supported the RT-qPCR findings, showing a significant reduction in the content of ZO-1, occludin, and claudin-3 following *T. spiralis* infection, with these levels reaching their minimum on 7 dpi (Figure 2B). The analysis of the expression changes of jejunal mucins (MUC-1, MUC-2), along with Toll-like receptors (TLR-1, TLR-2, and TLR-4), following *T. spiralis* infection, utilised similar methods. The results indicated that the expression levels of MUC-1 and MUC-2 underwent a marked reduction as early as 3 dpi (*P* < 0.001), reaching their minimal levels at 7 dpi (Figures 2C and D). Conversely, the expressions of TLR-1, TLR-2, and TLR-4 significantly augmented on 1 dpi (*P* < 0.01), peaking at 7 dpi (Figures 2E and F).

Furthermore, this study assessed the effects of *T. spiralis* infection on the expression of inflammatory cytokines (IL-1β, TNF-α, TGF-β, and IL-10). RT-qPCR and western blotting results showed a progressive escalation in the concentrations of TNF-α and IL-1β, commencing at 1 dpi (*P* < 0.01, *P* < 0.001, compared to 0 dpi) and culminating at 7 dpi, with a subsequent marked decrease by 15 dpi (compared to 7 dpi). Conversely, the expression of TGF-β and IL-10 exhibited a gradual decline from 1 dpi (*P* < 0.01, *P* < 0.001, compared to 0 dpi) to their lowest levels at 7 dpi. This decline was followed by a modest recovery at 15 dpi (compared to 7 dpi) (Figures 2G and H). These results collectively suggest that *T. spiralis* infection disrupts the barrier function of the jejunal mucosa.

### Isolation and purification of recombinant protein rKaSPI and rAdSPI

SPIs of *T. spiralis* play a pivotal role in its lifecycle. To investigate the effects of these SPIs on the intestinal mucosal barrier during *T. spiralis* invasion, the recombinant proteins rKaSPI and rAdSPI, which were previously preserved in our laboratory, were purified.

**Figure 3:**
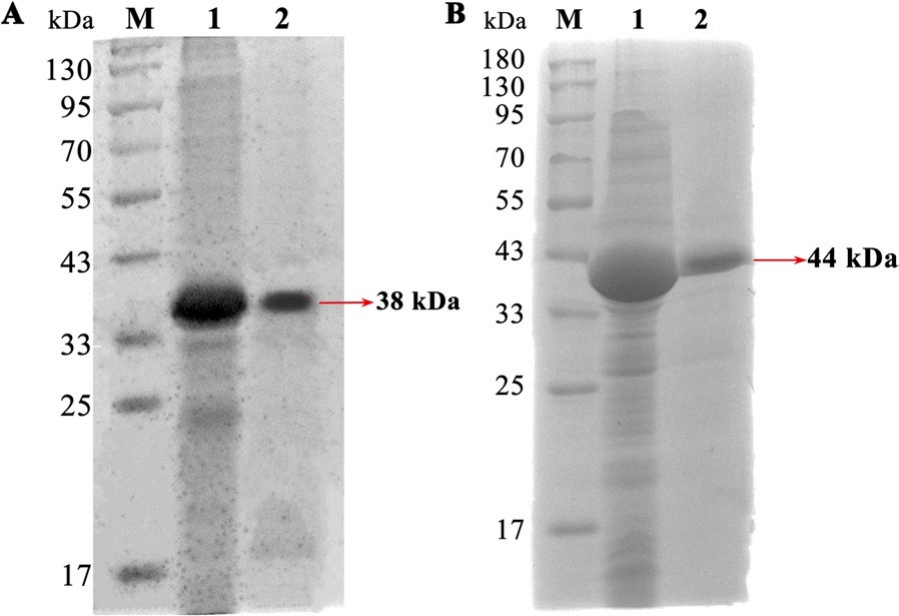
**Isolation and purification of recombinant protein rKaSPI and rAdSPI. A** Recombinant protein rKaSPI with a size of 38 kDa, and (**B**) Recombinant protein rAdSPI with a size of 44 kDa.

SDS-PAGE gel electrophoresis analysis showed that rKaSPI and rAdSPI displayed clear and precise single protein bands, aligning with the expected results. Specifically, the molecular weight of the rKaSPI protein was approximated at 38 kDa, whereas the rAdSPI protein was approximated at 44 kDa (Figure 3). These results verified the successful expression of both recombinant proteins and confirmed their accuracy and high purity.

### Effects of recombinant protein rKaSPI and rAdSPI on the barrier of IPECs

**Figure 4:**
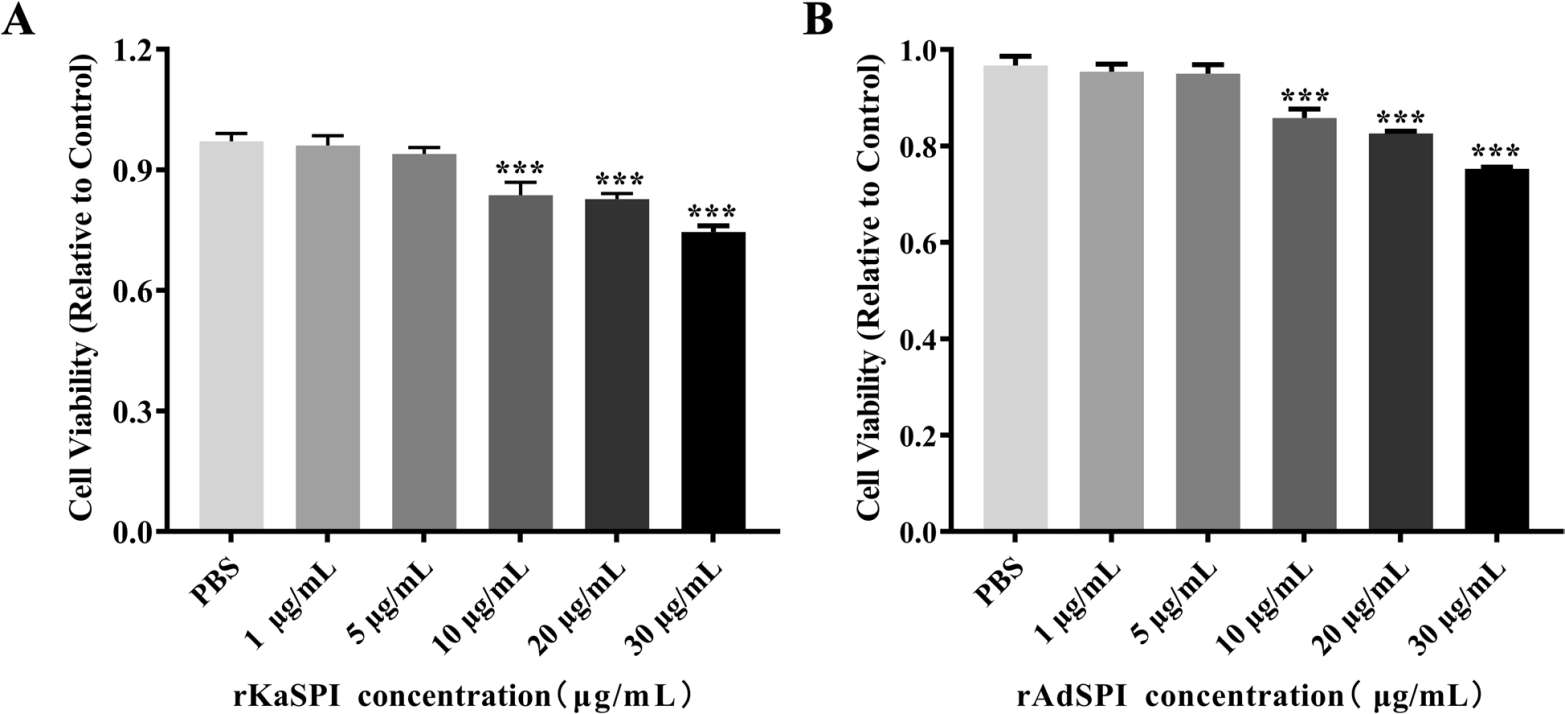
**Effects of rKaSPI and rAdSPI on IPEC viability.** IPECs were co-incubated with rKaSPI and rAdSPI for 24 h, respectively. The effects of rKaSPI (**A**) and rAdSPI (**B**) on cell proliferation were assessed using the CCK-8 assay at OD_450_ values. All assays were performed in triplicate, and data were presented as the mean ± SD. *P < 0.05, **P < 0.01, and ***P < 0.001 represented the significance levels of the comparisons between the PBS group and the treated groups.

An analysis was conducted of these two proteins for 24 h by co-culturing IPECs with varying concentrations (0, 1, 5, 10, 20, 30 μg/mL) to examine the impact of rKaSPI and rAdSPI on intestinal epithelial cell barrier function. Subsequently, the CCK8 assay was employed to assess their effects on IPEC proliferation. Notably, cell viability remained largely unaffected at concentrations below 5 μg/mL for both proteins. However, a marked decline in cell viability was detected when concentrations exceeded 10 μg/mL (*P* < 0.001) (Figure 4), leading to the determination of 10 μg/mL as the optimal working concentration for subsequent experiments involving both rKaSPI and rAdSPI.

**Figure 5:**
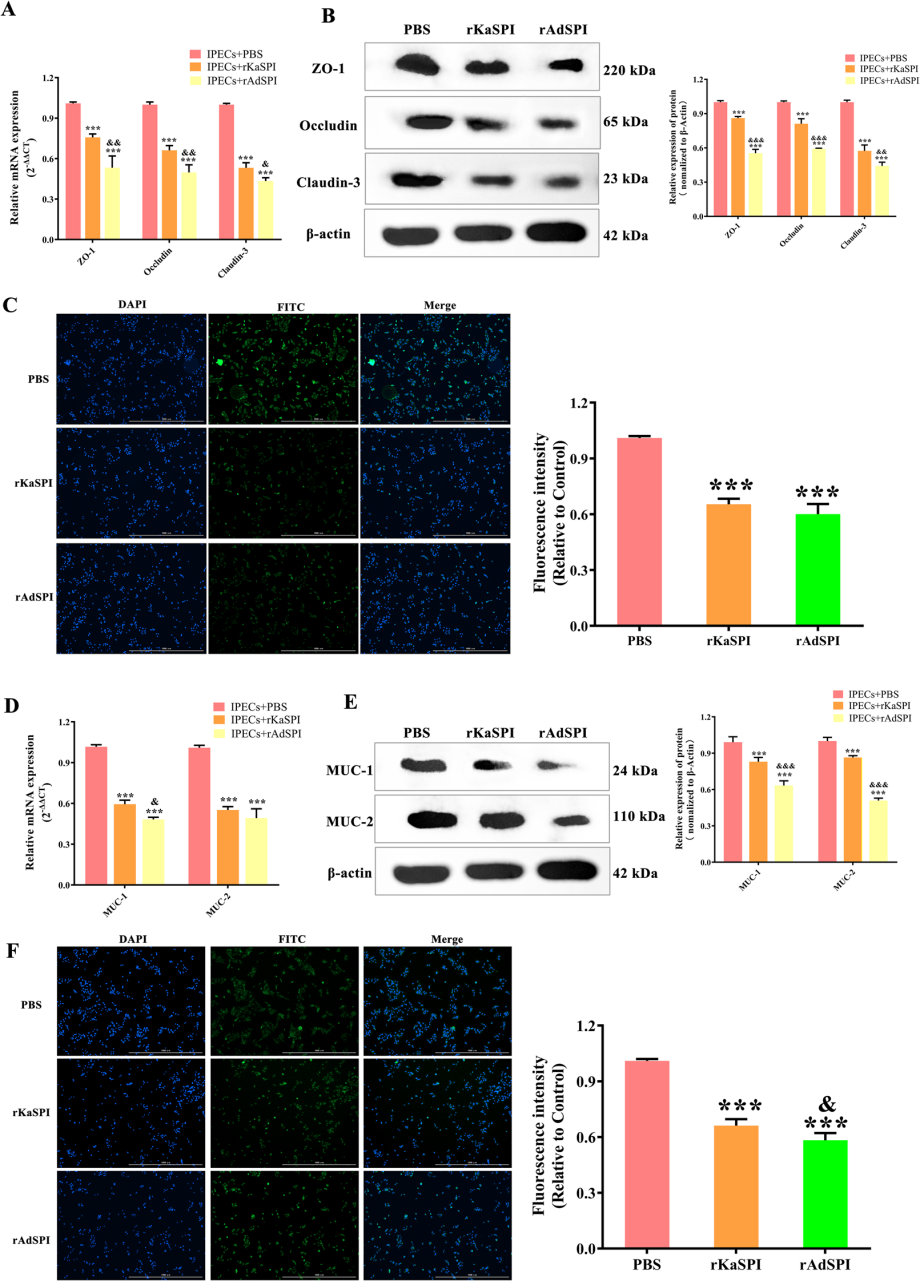
**Effects of rKaSPI and rAdSPI on TJ and Mucin expression in IPECs.** rKaSPI and rAdSPI were incubated with IPECs for 24 h, respectively, before carrying out the assays. **A** RT-qPCR, **B** Western blotting, and **C** Immunofluorescence (magnification: 4 × PL FL; scale bar: 1000 μm) analysed the expression of TJs in IPECs treated with rKaSPI and rAdSPI. **D** RT-qPCR, **E** Western blotting and **F** Immunofluorescence (magnification: 4 × PL FL; scale bar: 1000 μm) analysed the expression of Mucins in IPECs treated with rKaSPI and rAdSPI. Assays were performed in triplicate, and the data were expressed as the mean ± SD. *P < 0.05, **P < 0.01, ***P < 0.001 compared with the IPECs + PBS group; &*P* < 0.05, &&*P* < 0.01, &&&*P* < 0.001 compared with the IPECs + rKaSPI group.

An interaction model between rKaSPI/rAdSPI and IPECs was established to elucidate their mechanisms further, focusing on alterations in TJ proteins, mucins, TLRs, and inflammatory cytokines. RT-qPCR and western blotting analyses exposed a significant down-regulation of ZO-1, occludin, and claudin-3 mRNA and protein levels in both IPECs + rKaSPI and IPECs + rAdSPI groups compared to the untreated control (IPECs + PBS). Notably, the inhibitory effect was more pronounced in the IPECs + rAdSPI group (*P* < 0.001, compared to the rKaSPI group), especially regarding occludin and claudin-3, as indicated by more significant declines in their mRNA and protein expressions (Figures 5A and B). This observation was further supported by immunofluorescence assays targeting claudin-3, which exhibited markedly reduced fluorescence intensity in both treatment groups (Figure 5C), consistent with the RT-qPCR and western blotting results.

**Figure 6:**
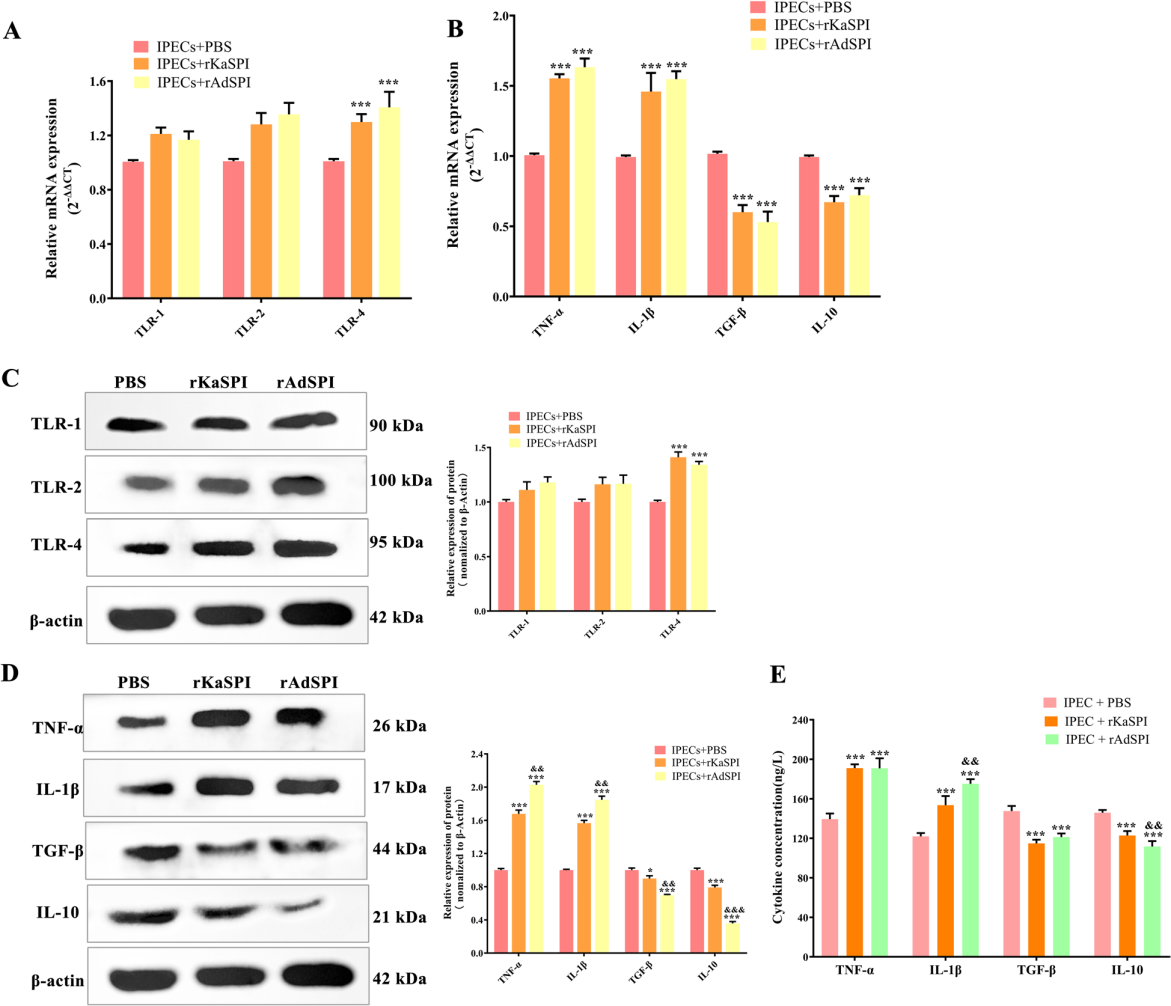
**Effects of rKaSPI and rAdSPI on TLR and inflammatory cytokine expression in IPECs. A** RT-qPCR and **C** Western blotting analysed the expression of TLRs in IPECs treated with rKaSPI and rAdSPI. **B** RT-qPCR, **D** Western blotting and **E** ELISA analysed the expression of inflammatory cytokines in IPECs treated with rKaSPI and rAdSPI. Assays were performed in triplicate, and the data were expressed as the mean ± SD. Significance markers were the same as those in Figure 5.

Furthermore, the expression of mucins MUC-1 and MUC-2 was significantly down-regulated (Figures 5D and E), whereas TLR-4 expression increased considerably in both treated groups (*P* < 0.001), with no noticeable changes in TLR1 or TLR-2 levels (Figures 6A and C). Immunofluorescence of MUC-2 corroborated these findings, demonstrating a substantial decrease in fluorescence intensity post-treatment (Figure 5F). This outcome was also consistent with the RT-qPCR and western blotting results.

Given the intricate interplay between inflammatory cytokines and the function of the intestinal epithelial barrier, this study also investigated the expression of TNF-α, IL-1β, TGF-β, and IL-10 in IPECs following treatment with rKaSPI and rAdSPI. We systematically analysed these cytokines using RT-qPCR, western blotting, and sandwich ELISA.

The results showed that both rKaSPI and rAdSPI significantly up-regulated the mRNA and protein levels of pro-inflammatory cytokines TNF-α and IL-1β (*P* < 0.001) while down-regulating those of anti-inflammatory cytokines TGF-β and IL-10 (*P* < 0.05, *P* < 0.001). Remarkably, the up-regulation of TNF-α was more pronounced in the IPECs + rAdSPI group compared to the IPECs co-cultured with rKaSPI (Figures 6B and D). The ELISA analysis of the cell culture supernatants supported the findings from RT-qPCR and western blotting (Figure 6E). These results confirm that rKaSPI and rAdSPI play a regulatory role in the expression of inflammatory cytokines in IPECs.

### Effects of recombinant protein rKaSPI and rAdSPI on the intestinal barrier of the host

**Figure 7:**
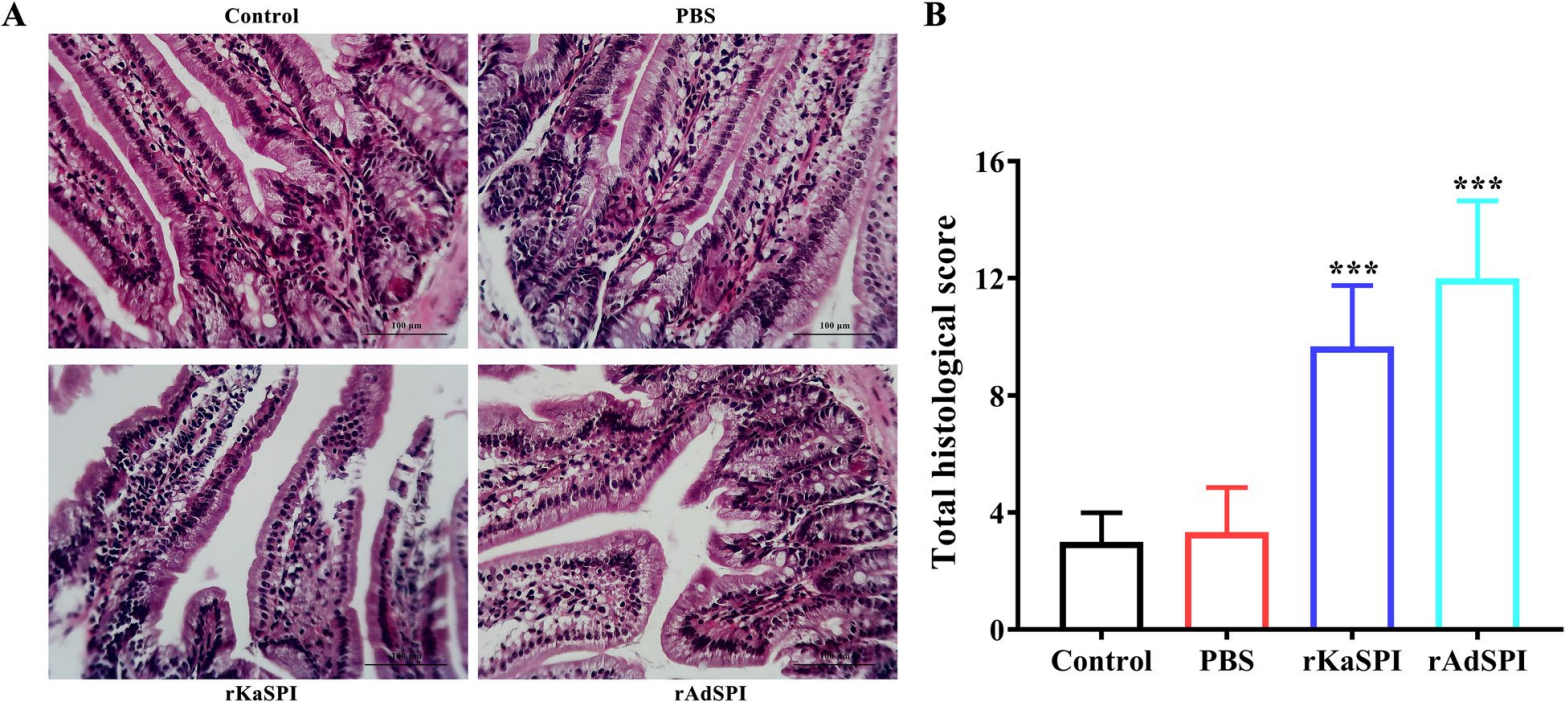
**H&E staining evaluation of pathological injury of rKaSPI and rAdSPI to the jejunum.** Histopathological sections of the jejunum were collected from mice on the third day post-injection of rKaSPI and rAdSPI, respectively. **A** H&E staining of the jejunum. Magnification, × 200. **B** The intestinal pathology scores. The data were expressed as the mean ± SD. *P < 0.05, **P < 0.01, ***P < 0.001 compared with the PBS group, and &*P* < 0.05, &&*P* < 0.01, &&&*P* < 0.001 compared with the rKaSPI group.

A mouse trial was designed and executed to clarify the specific effects of recombinant proteins rKaSPI and rAdSPI on the host’s intestinal mucosal barrier function. A pathological assessment of jejunal tissue sections showed a notable loss of jejunal villus in mice subjected to rKaSPI and rAdSPI injections compared to the untreated control group. Furthermore, we observed a significant infiltration of inflammatory cells within the jejunal tissues (Figure 7A). A double-blinded pathological scoring system was applied to jejunal tissues from all groups to quantify the extent of pathological damage. The results unequivocally demonstrated a statistically significant elevation in lesion scores in the rKaSPI- and rAdSPI-treated groups compared to the control group (Figure 7B).

**Figure 8:**
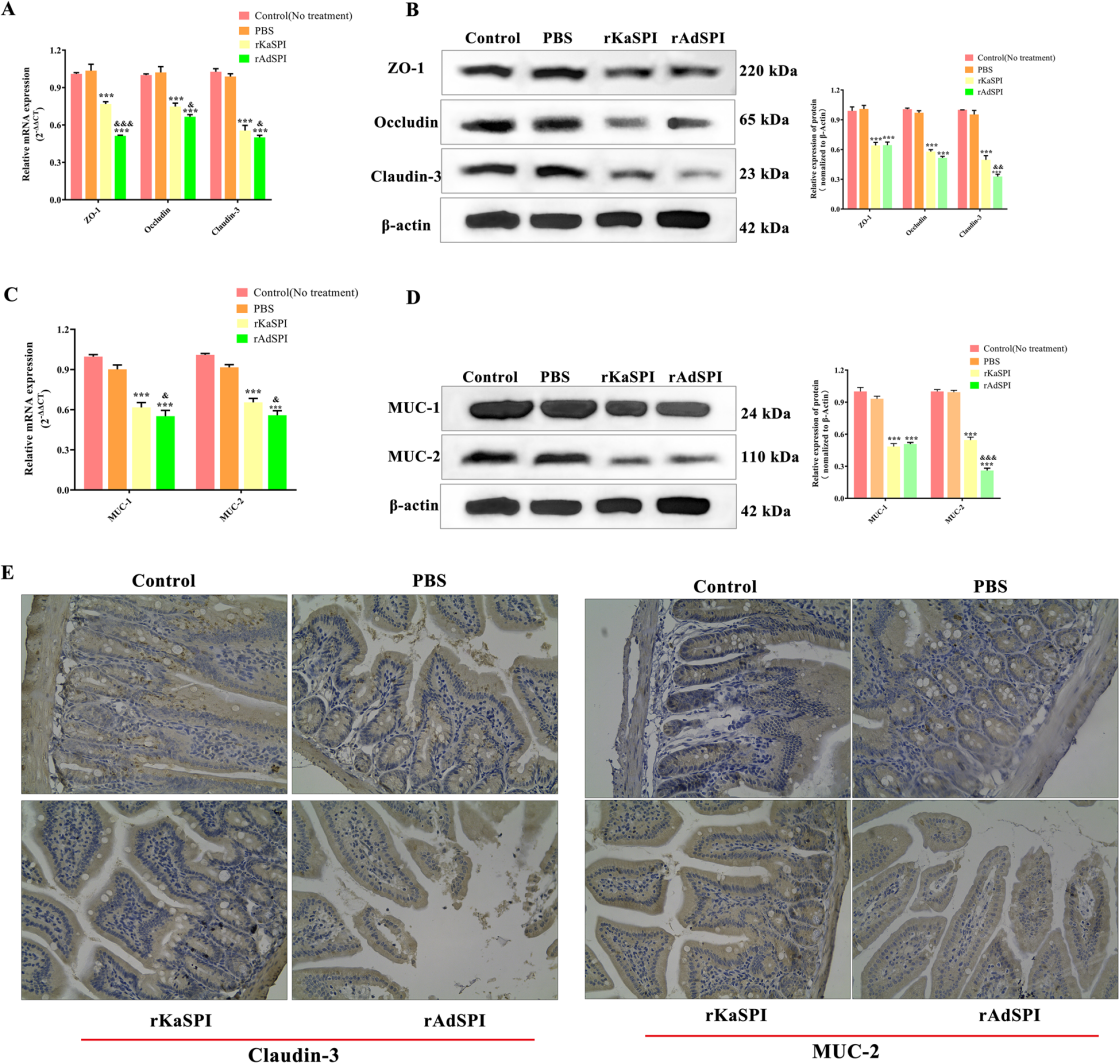
**Effects of rKaSPI and rAdSPI on TJ and Mucin expression of the jejunum.** The assays began three days after the mice were intraperitoneally injected with rKaSPI and rAdSPI. Verification of expression changes of TJ proteins (**A**, **B**) and mucins (**C**, **D**) by RT-qPCR and western blot, respectively. (**E**) Using immunohistochemistry to verify expression changes of claudin-3 and MUC-2. Assays were performed in triplicate, and the data were expressed as the mean ± SD. *P < 0.05, **P < 0.01, ***P < 0.001 compared with the PBS group; &*P* < 0.05, &&*P* < 0.01, &&&*P* < 0.001 compared with the rKaSPI group.

To gain a deeper understanding of the barrier function and its molecular regulatory mechanisms, we utilised RT-qPCR and western blotting to assess the effects of rKaSPI and rAdSPI on the expression levels of ZO-1, occludin, and claudin-3 in jejunal tissues. The results showed that recombinant proteins significantly down-regulated the transcription and translation of ZO-1, occludin, and claudin-3 (Figures 8A and B). The reduction in claudin-3 expression was confirmed by immunohistochemistry analysis, which showed a brownish-yellow staining pattern primarily located at the lateral membranes of IPECs at the edges of the villi. In the rKaSPI and rAdSPI groups, inflammatory infiltrates and adjacent oedematous regions decreased markedly (Figure 8E, left), further supporting RT-qPCR and western blotting findings.

**Figure 9:**
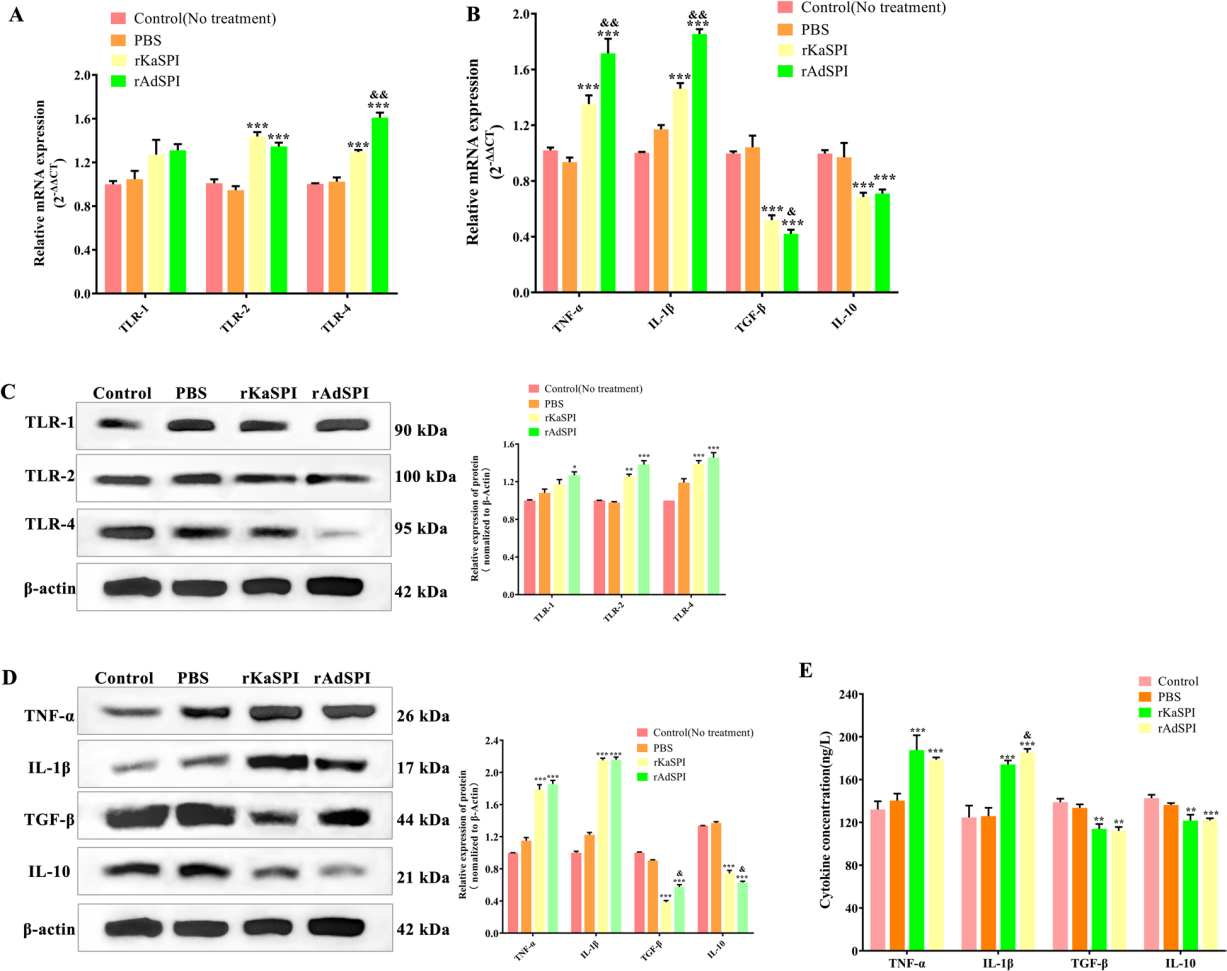
**Effects of rKaSPI and rAdSPI on TLR and inflammatory cytokine expression of the jejunum. A** RT-qPCR and **C** Western blotting analysed the expression of TLRs in the jejunum. **B** RT-qPCR, **D** Western blotting, and **E** ELISA analysed the expression of inflammatory cytokines in the jejunum. Assays were performed in triplicate, and the data were expressed as the mean ± SD. Significance markers were the same as those in Figure 8.

The influence of rKaSPI and rAdSPI on jejunal mucins (MUC-1, MUC-2) and Toll-like receptors (TLR-1, TLR-2, and TLR-4) was also evaluated. RT-qPCR and western blotting analyses indicated that both proteins effectively decreased MUC-1 and MUC-2 expression (Figures 8C and D) while significantly up-regulating the expression of TLR2 and TLR4 (Figures 9A and C). Immunohistochemistry of MUC-2 further confirmed its reduced expression in the rKaSPI and rAdSPI groups (Figure 8E, right). Notably, rAdSPI exhibited a more pronounced effect on the expression of TJ proteins (claudin-3) and mucins (MUC-2) compared to rKaSPI.

To investigate the immunomodulatory roles of rKaSPI and rAdSPI, the expression of TNF-α, IL-1β, TGF-β, and IL-10 in jejunal tissues and serum was systematically analysed using RT-qPCR, western blotting, and sandwich ELISA. The two recombinant proteins significantly up-regulated TNF-α and IL-1β expression while down-regulating TGF-β and IL-10 in jejunal tissues (Figures 9B and D), corroborating ELISA results (Figure 9E). Notably, the rAdSPI injection group produced significantly lower levels of IL-10 at translational levels in jejunal tissues compared to the rKaSPI injection group.

## Discussion

The invasion process of *T. spiralis* is highly complex and multilayered, encompassing parasitic life cycles, immune evasion mechanisms, molecular interactions, and genetic regulation. Dissecting the invasion mechanisms of *T. spiralis* can provide critical insights into the pivotal steps during infection. Throughout evolution, *T. spiralis* has developed diverse invasion strategies, particularly leveraging its ESPs, which play a central role in inter-parasite communication and in interacting with host cells and evading host immune rejection [22, 23]. This study focuses on a key component of the ESPs of *T. spiralis* (SPIs), aiming to unravel their potential roles in the parasite’s breach of the host’s intestinal mucosal barrier and the successful establishment of parasitism.

IECs constitute the first line of physical defence in the gut, safeguarding against luminal antigens, toxins, and harmful substances while actively participating in intestinal mucosal immune responses [24]. They are also the crucial juncture that *T. spiralis* must traverse during invasion.

Our study initially probed into the impact of *T. spiralis* invasion on intestinal mucosal barrier function. We established an infection model to evaluate the pathological changes in the jejunum of mice infected with *T. spiralis* at various days post-infection (0, 1, 3, 7, 15 dpi). The results indicated that marked jejunal tissue lesions, characterised by extensive villus exfoliation and disruption accompanied by a substantial infiltration of inflammatory cells, were evident at 3 dpi. Moreover, a double-blinded pathological scoring system revealed that lesion scores peaked at 7 dpi, aligning with the parasitic life cycle within the host. These findings suggest that this time point represents the peak of *T. spiralis*-induced intestinal tissue damage.

It is well-known that TJs, as crucial intercellular structures, are indispensable for maintaining the functional integrity and stability of the intestinal epithelial barrier [25]. Their functionality relies on intricate and precise interactions among a series of TJ proteins, including members of the claudin, zonula occludens, and tight junction-associated MARVEL protein (TAMP) families [26]. Numerous studies have demonstrated that parasites and their products can directly interact with IECs, modulating the expression of TJ proteins, thereby facilitating parasite intracellular parasitism [27, 28].

Therefore, this study explored the effects of *T. spiralis* infection on TJ proteins in the host jejunum at different time points. The results revealed that *T. spiralis* infection significantly down-regulated the expression of several key TJ proteins (ZO-1, occludin, and claudin-3) in the host jejunum. Specifically, ZO-1 expression decreased significantly early in infection (at 1 dpi), whereas occludin and claudin-3 levels significantly declined from 3 dpi, reaching their nadir at 7 dpi. This outcome indicates that *T. spiralis* infection gradually disrupts the intestinal mucosal barrier, with the most severe damage occurring within the first week of post-infection.

TLRs are an integral part of the innate immune system and are widely expressed in IECs, immune cells, and other cell types in the gut. They are crucial for maintaining intestinal barrier function and regulating immune responses [29]. Additionally, mucins exert multifaceted effects on the IEC barrier function, including enhancing chemical barriers, maintaining physical barriers, and modulating immune responses. Collectively, these effects safeguard intestinal homeostasis and immunity [30]. Furthermore, the study investigated the regulatory effects of *T. spiralis* infection on two crucial innate immune modalities within the intestinal barrier: TLRs and mucins.

Our results showed that MUC-1 and MUC-2 expression levels significantly decreased from 1 dpi, with this downward trend persisting until 7 dpi when levels reached their lowest expression. This result suggests that *T. spiralis* infection may impair the mucin barrier of the intestinal mucosa, inhibiting MUC-1 and MUC-2 expression. Interestingly, this study demonstrated that *T. spiralis* infection led to the proliferation of goblet cells in the host, which were primarily responsible for producing MUC. Similarly, IPECs also contributed to MUC production to some extent. However, the results indicated a decrease in overall mucin levels in the jejunum.

It was hypothesised that this reduction was due to the predominance of IECs in the jejunum, which ultimately decreased overall MUC levels. In contrast, the expression levels of TLR-1, TLR-2, and TLR-4 exhibited an inverse trend compared to mucins. Notably, although the study observed up-regulated Toll-like receptor expression, the authors’ previous work found that *T. spiralis* exosomes possessed the capacity to down-regulate Toll-like receptor expression [31]. This interesting contrast hints at the existence of complex immune regulatory mechanisms during *T. spiralis* infection, which merits further investigation.

Inflammatory factors impact the integrity and permeability of the intestinal barrier through various mechanisms. For instance, inflammatory factors such as TNF-α and IL-1β can affect TJs in IECs, thereby increasing their permeability [32]. Conversely, IL-10 protects the intestinal epithelial barrier function from damage by inhibiting the production and activity of inflammatory cytokines [33].

The intensification of inflammation that we observed in histological sections of the jejunum post-*T. spiralis* infection was closely related to changes in the expression of inflammatory cytokines. Analysis revealed that TNF-α and IL-1β, important pro-inflammatory cytokines, gradually increased on different days post-infection (1, 3, 7, 15 dpi), peaking at 7 dpi. This finding suggests that the host’s inflammatory response gradually intensifies as the infection progresses to counter the invasion of *T. spiralis*.

However, the expression levels of TNF-α and IL-1β decreased significantly at 15 dpi, compared to 7 dpi, potentially indicating the onset of the resolution phase of the inflammatory response. In contrast to pro-inflammatory factors, anti-inflammatory cytokines such as TGF-β and IL-10 exhibited a gradual decline in expression during the initial stages of infection, with a recovery observed at 15 dpi. This outcome suggests that as the infection is gradually controlled, the host’s anti-inflammatory mechanisms begin to restore and function, balancing and regulating the immune response and thus promoting tissue repair and restoration.

SPIs are essential components of *T. spiralis* ESPs and possess unique biological functions. Previous studies in our laboratory definitively confirmed that when *T. spiralis* invades the host, its secreted SPIs can trigger a series of significant phenotypic changes in host cells, such as substantially enhancing endoplasmic reticulum stress [17] and initiating and enhancing autophagy [34]. Subsequently, this study constructed in vitro models of the interaction between two recombinant proteins (rKaSPI and rAdSPI) and IPECs to further establish in vivo models of their effects in mice.

The study aimed to thoroughly elucidate the specific mechanisms by which rKaSPI and rAdSPI influence intestinal barrier function by assessing their impact on the expression of TJs, mucins, TLRs, and inflammatory cytokines in IPECs and mouse jejunum tissues. Results indicated that both rKaSPI and rAdSPI exhibited regulatory effects on specific gene expression in both cellular and animal models. They collectively down-regulated the expression levels of TJ proteins (e.g., ZO-1, occludin, claudin-3), mucins (MUC-1, MUC-2), and anti-inflammatory factors (TGF-β, IL-10), while up-regulating the expression of TLR-4 and pro-inflammatory factors (IL-1β, TNF-α). This pattern of changes was highly consistent with the observed gene expression changes in the jejunum tissues of mice infected with *T. spiralis*. As such, the outcome emphasises the crucial roles of rKaSPI and rAdSPI in the invasion process of *T. spiralis* and their significant impact on intestinal mucosal barrier function.

Further observations also found that while both rKaSPI and rAdSPI promoted TLR-4 expression, *T. spiralis* infection also involves the regulation of TLR-1 and TLR-2 expression, highlighting the complexity of the parasite’s infection mechanism. When comparing the specific effects of rKaSPI and rAdSPI on gene expression, rAdSPI exhibited a more pronounced regulatory effect. This may be related to the specificity of different life cycle stages of *T. spiralis*. Additionally, as AdSPI originates from adult worms parasitising the small intestine and KaSPI comes from muscle larvae parasitising muscle tissue, this difference may explain the stronger efficacy of rAdSPI in influencing small intestine-related gene expression.

In summary, *T. spiralis* infection in the host led to jejunal tissue lesions, accompanied by significant decreases in the expression of TJ proteins (e.g., ZO-1, occludin, claudin-3) and mucins (MUC-1, MUC-2), along with up-regulated Toll-like receptor expression and intensified inflammatory responses.

Further in-depth research revealed that two important proteins secreted by *T. spiralis* (KaSPI and AdSPI) played pivotal roles in this process. By reducing the TJ proteins and mucin levels, they disrupted the TJ state of the host intestine. Subsequently, they affected innate immune responses, promoted the expression of TLRs, and facilitated the release of inflammatory factors (e.g., IL-1β, TNF-α), ultimately weakening the intestinal mucosal barrier function.

The study clarifies the specific roles of KaSPI and AdSPI in the infection mechanism of *T. spiralis* and provides valuable insights into how the parasite successfully invades the host.

## Data Availability

The datasets used or analysed during the current study are available from the corresponding author upon reasonable request.
